# Bioprospecting Plant-Derived Natural Products: Integrating Chemical Diversity, Efficacy, Safety, and Technological Development

**DOI:** 10.3390/plants15162468

**Published:** 2026-08-14

**Authors:** Rosy Iara Maciel de A. Ribeiro, Renê Oliveira do Couto

**Affiliations:** 1 Laboratory of Experimental Pathology (LAPATEX), Federal University of São João del-Rei, Midwest Campus (Dona Lindu), St. Sebastião Gonçalves Coelho 400, Chanadour, Divinópolis 35501-296, MG, Brazil; 2 Laboratory of Pharmaceutical Development (LADEF), Federal University of São João del-Rei, Midwest Campus (Dona Lindu), St. Sebastião Gonçalves Coelho 400, Chanadour, Divinópolis 35501-296, MG, Brazil; rocouto@ufsj.edu.br

The study of plant-derived natural products remains one of the most promising avenues for discovering new bioactive agents. Natural products have provided essential lead compounds and therapeutic agents that have shaped modern drug discovery and development [1,2]. Plants are an extraordinary source of chemical diversity, largely because of their ability to biosynthesize a wide array of secondary metabolites with distinct biological functions and significant pharmacological potential [3].

These metabolites have been associated with a broad spectrum of biological activities, including antioxidant, antimicrobial, antiviral, anti-inflammatory, and anticancer effects, reinforcing their relevance across pharmaceutical, biomedical, cosmetic, and agricultural applications [3,4]. However, contemporary bioprospecting extends far beyond identifying biological activities. It requires rigorous phytochemical characterization, extract standardization, pharmacological validation, toxicological assessment, and formulation development to ensure quality, reproducibility, efficacy, stability, and safety [2,5].

Owing to its inherent complexity and multidisciplinary character, natural product research demands strong collaboration among research groups. The challenges associated with this field cannot be effectively addressed in isolation; rather, they require the integration of diverse expertise, technologies, and strategic approaches. Thus, collaborative networks are fundamental to strengthening the field and accelerating the translation of discoveries into practical applications. We hope that this Special Issue also fosters collaboration and integration among research groups worldwide.

The articles published in this Special Issue clearly reflect the breadth and growing maturity of this field. The study on the standardized spray-dried *Celtis iguanaea* (Cannabaceae) leaf extract provides important evidence supporting its potential for managing acute pain and inflammation, illustrating how traditionally used plant species can be systematically investigated through integrated experimental and pharmaceutical approaches [6].

Complementing this perspective, the study evaluating the subchronic toxicity and cognitive effects of the methanolic extract of *Micromeria frivaldszkyana* (Lamiaceae) reinforces a key principle for the rational use of phytomedicines: medicinal herbs should not be presumed safe solely by dint of their natural origin. Systematic toxicological evaluation is essential for defining safety margins and guiding future therapeutic applications [7].

The relationship between chemical composition and biological activity is also highlighted in the study on leaf and bark extracts from *Sarcomphalus joazeiro* (Rhamnaceae), in which phenolic compounds were quantified via HPLC-DAD and associated with antioxidant, antileishmanial, and cytotoxic activities [8]. This approach is particularly valuable because it directly links chemical composition to functional validation, thereby enhancing the reproducibility and reliability of the findings.

Similarly, the study on spray-dried extracts of *Lippia sidoides* (Verbenaceae) expands the pharmacological potential of this species by demonstrating anticonvulsant, anticholinesterase, and cytoprotective effects, highlighting its potential for application in neurological disorders [9].

The cosmetic potential of natural products is also represented in the study on *Gynostemma pentaphyllum* (Cucurbitaceae) extracts prepared using natural deep eutectic solvents and ultrasound-assisted extraction [10]. This work highlights an important trend in the field, i.e., the adoption of more sustainable, efficient, and industry-compatible extraction methods. In the same direction, the study on *Teucrium chamaedrys* (Lamiaceae) demonstrates antioxidant and anti-melanogenesis effects in B16-F10 cells, bringing phytochemistry closer to current demands in dermatology, photoprotection, and cosmeceutical development [11].

Another important area of innovation lies in phytopharmaceutical technology. The development of 3D-printed tablets containing *Mucuna pruriens* (Fabaceae) seed extracts, guided by experimental design and multivariate statistical analysis, illustrates how bioprospecting must extend beyond biological screening [12]. For natural products to become viable therapeutic interventions, critical attributes such as dose control, release profiles, stability, acceptability, and overall pharmaceutical performance must be carefully addressed. In the same context, the use of scalable unit operations—such as spray-drying—and the development of optimized delivery systems reinforce the notion that product quality should be considered from the earliest stages of research [6,12], in accordance with Quality by Design (QbD) principles widely cited in the pharmaceutical industry [13].

The diverse topics in this Special Issue also include agroecological applications, as shown by the study on the allelopathic potential of *Artemisia absinthium* (Asteraceae) and *Artemisia vulgaris* (Asteraceae) from Serbia [14]. By evaluating their chemical composition and bioactivity against weeds, this article expands the concept of bioprospecting beyond human health and highlights sustainable alternatives for agricultural management.

The study on converting phenolic monoterpenes from the essential oil of *Conobea scoparioides* (Plantaginaceae) using hydrotalcite synthesized from blast-furnace slag further highlights the intersection between natural product chemistry, catalysis, and the use of industrial residues, reinforcing the technological and sustainable dimensions of this research area [15].

Although oncology remains one of the most challenging areas in drug discovery and pharmaceutical development [16], it is also a major focus of industrial pipelines and R&D investment [17]. Natural products are uniquely positioned within this landscape, offering structurally diverse scaffolds and novel mechanisms of action. In this context, this Special Issue highlights advances in the field through two complementary review articles that address the therapeutic potential of natural products from distinct perspectives.

Melo et al. examine cannabidiol as a bioactive compound with antitumor activity across multiple tumor models, with an emphasis on mechanisms of cell death [18]. An et al. focus on natural products for melanoma therapy, highlighting the transition from traditional medicine to modern drug discovery [19]. Together, these contributions illustrate how natural compounds can act not only as cytotoxic agents but also as modulators of key cellular processes involved in tumor progression, survival, and therapeutic responses.

Taken together, the contributions to this Special Issue demonstrate that research on plant-derived natural products is increasingly driven by an integrated, translational framework that encompasses identification, characterization, biological validation, analytical standardization, and formulation. This paradigm is essential to overcome long-standing challenges in the field, such as chemical complexity and variability, limited reproducibility, insufficient toxicological data, and overinterpretation of traditional use. Therefore, evidence-based bioprospecting requires botanical, chemical, pharmacological, technological, and regulatory rigor.

Overall, this collection of articles demonstrates that plant-derived natural products continue to play a strategic role in the discovery of new bioactive compounds and in the development of therapeutic, cosmetic, veterinary, and agroecological solutions. Beyond summarizing the contributions gathered in this Special Issue, this editorial highlights a necessary direction for the field: transitioning from exploratory bioprospecting to an integrated, evidence-based, and translational framework. By uniting phytochemistry, pharmacology, toxicology, pharmaceutical technology, and sustainability, this Special Issue stresses that the future of plant-derived natural products depends not only on discovering bioactivity but also on transforming biological and chemical diversity into reproducible, safe, effective, and socially impactful innovations.
